# Two- to Six-year Assessment of Survivorship and Patient-Reported Outcomes in Rotating Hinge Knee Prostheses With Porous Tibial Cone Augmentation

**DOI:** 10.1016/j.artd.2025.101724

**Published:** 2025-06-02

**Authors:** Matthew Van Engen, Taylor Den Hartog, Natalie Glass, Nicolas Noiseux

**Affiliations:** Department of Orthopedics and Rehabilitation, University of Iowa Hospitals and Clinics, Iowa City, IA, USA

**Keywords:** Rotating hinge, Hinge knee, Tibial metaphyseal cone, Revision TKA, Outcomes

## Abstract

**Background:**

Rotating hinge knee (RHK) implants are a highly constrained prosthesis designed for knee arthroplasty cases involving significant knee instability. RHK systems are often augmented with metaphyseal tibial cones (MTCs). This study investigated structural stability and patient-reported outcomes (PROs) of RHKs with MTC augmentation.

**Methods:**

All RHKs utilizing MTC were identified at a single institution from 2016 to 2021. Patients returned for radiographic evaluation, physical exam, and PROs. A total of 84 knees (80 patients) were identified, 44 cases (43 patients) followed up greater than 2 years after their RHK procedure. A cumulative incidence function curve was constructed, and failure was defined as a subsequent revision. PROs included Knee Injury Osteoarthritis Outcome Score for Joint Replacement and Patient-Reported Outcome Measurement Information System (PROMIS)−10. Patient Acceptable Symptom State and postoperative rating of improvement scores were collected.

**Results:**

Cumulative incidence function all-cause failure (N = 84) was 18% at 2.8 years. The diagnoses for failed cases were infection (6 cases), mechanical failure (1 case), and persistent pain (1 case). The mechanical failure was due to loosening of the femoral component requiring femoral only revision. No follow-up cases (N = 44) demonstrated evidence of loosening at the MTC–RHK interface, median follow-up time of 3.82 years. Mean range-of-motion was 0°-120°. Mean PROs were 61.4 for Knee Injury Osteoarthritis Outcome Score for Joint Replacement, 41.2 for PROMIS Mental Health, and 47.9 for PROMIS Physical Health. Patient Acceptable Symptom State was attained by 68% of follow-up cases and 75% reported moderate or better improvement in their quality of life after their RHK.

**Conclusions:**

RHK with MTC augmentation did not reveal any cases of loosening of the MTC with only 1 case of femoral loosening.

**Level of Evidence:**

III.

## Introduction

Rotating hinge knee (RHK) implants are a highly constrained subset of total knee arthroplasty (TKA). RHKs have relatively specific indications in primary arthroplasty and are reserved for complex cases such as severe angular deformity, instability, and neuromuscular disease [[1], [2], [3], [4]]. Hinge knee arthroplasty has previously been referred to as a salvage procedure and was used in the more complex revision cases as a last resort before arthrodesis or amputation [5]. RHKs are a way to address generalized soft tissue deficiency and fusion take down in lower demand patients. [3,6]. Additionally, RHK can address larger bone defects which often accompany these complex arthroplasty cases [7,8]. Knee arthroplasty cases with diminished bone stock often utilize porous metaphyseal cones. There has been a growing body of literature supporting the use of metaphyseal cones in revision TKA cases with revision-free survival rates reaching greater than 90% [9]. Questions accompany the structural stability of the metaphyseal tibial cone (MTC) when it is paired with a RHK given hinge knee implants increase stress forces at the bone–cement interfaces [10,11]. Aseptic loosening is one of the leading causes of hinge prosthesis failure along with periprosthetic joint infection [12,13]. To our knowledge, evidence is scarce regarding the investigation of both the structural durability and patient outcomes of a hinge knee prosthesis with tibial cone augmentation. Additionally, the limited studies pertaining to hinge knee arthroplasty in general consist of smaller sample sizes [4].

The primary purpose of this study was to examine the durability and survivorship of the RHK when augmented with a MTC. The secondary aim was to describe patient-reported outcome (PRO) scores in these patients.

We hypothesized the structural stability of a RHK when combined with MTC would result in a lower rate of revision for aseptic loosening in comparison to previous reports of RHK with no augmentation due to enhanced fixation of the hinge tibial component [13,14].

## Material and methods

This study was approved by our institutional review board. All patients who received a primary or revision RHK between January 1, 2016, and December 31, 2022, were identified using their respective current procedural terminology codes (27445 and 27487). Inclusion criteria for the study cohort were age >18 years and primary or revision RHK with MTC augmentation. Femoral cone placement was not required for inclusion in the study. Retrospective chart review was performed and demographic information such as age, sex, and contact information was obtained in addition to body mass index, history of diabetes, and smoking status at the time of surgery. Death records were reviewed and living patients were sent the study information via email if an email address was on file or physical mail if no email was listed in their medical record. An informed consent document was also included with the study information. If patients elected to participate, they were offered the opportunity to be seen in clinic and have radiographs taken of their knee in accordance with standard 1-, 2-, and 5-year follow-up appointments with further appointments occurring every subsequent 5 years. If the patient had already been seen for their latest routine follow-up, they were not required to be seen in clinic and the most recent radiographs and clinical notes were reviewed. Postoperative radiographs, collected at the patient’s latest follow-up clinical visit >2 years, were analyzed to assess the structural stability of the RHK with MTC construct. Additionally, active range of motion was recorded with a goniometer. Survivorship was assessed in 2 ways: first being all cause survivorship, where failure was defined as any reoperation for the RHK and second being loosening or failure, specifically at the tibial component-cone construct. Additional chart review was conducted to assess for further operations performed on the patient’s hinge knee, and patients were contacted to determine whether they had further procedures performed that may not be listed in our medical record.

In addition to following up in clinic, patients were sent PRO forms including Patient-Reported Outcome Measurement Information System (PROMIS)−10 and Knee Injury Osteoarthritis Outcome Score for Joint Replacement (KOOS-JR), a measure that describes knee functional limitations, pain and symptoms. Participants responded “Yes” or “No” to the question: “Considering your daily pain, function, and activity, is the current state of your knee acceptable?” to determine whether a Patient Acceptable Symptom State (PASS) was achieved at postoperative follow-up. Participants also rated their level of preoperative to postoperative improvement on a scale of 1-5 corresponding with “No improvement”, “Little improvement”, “Moderate improvement”, “Great improvement” or “More improvement than I thought possible” in response to the question “How has your knee surgery impacted your quality of life?”. Patient follow-up in clinic was not required for completion of the outcome forms. PRO forms were obtained via email with a link connecting the patients to the survey in REDCap, which is an electronic data capture tool hosted at the University of Iowa Hospitals and Clinics, Iowa City, Iowa [15,16]. Patients without an email address on file were sent physical copies of the PRO surveys via mail using the provided home address listed in their medical record.

**Figure 1:**
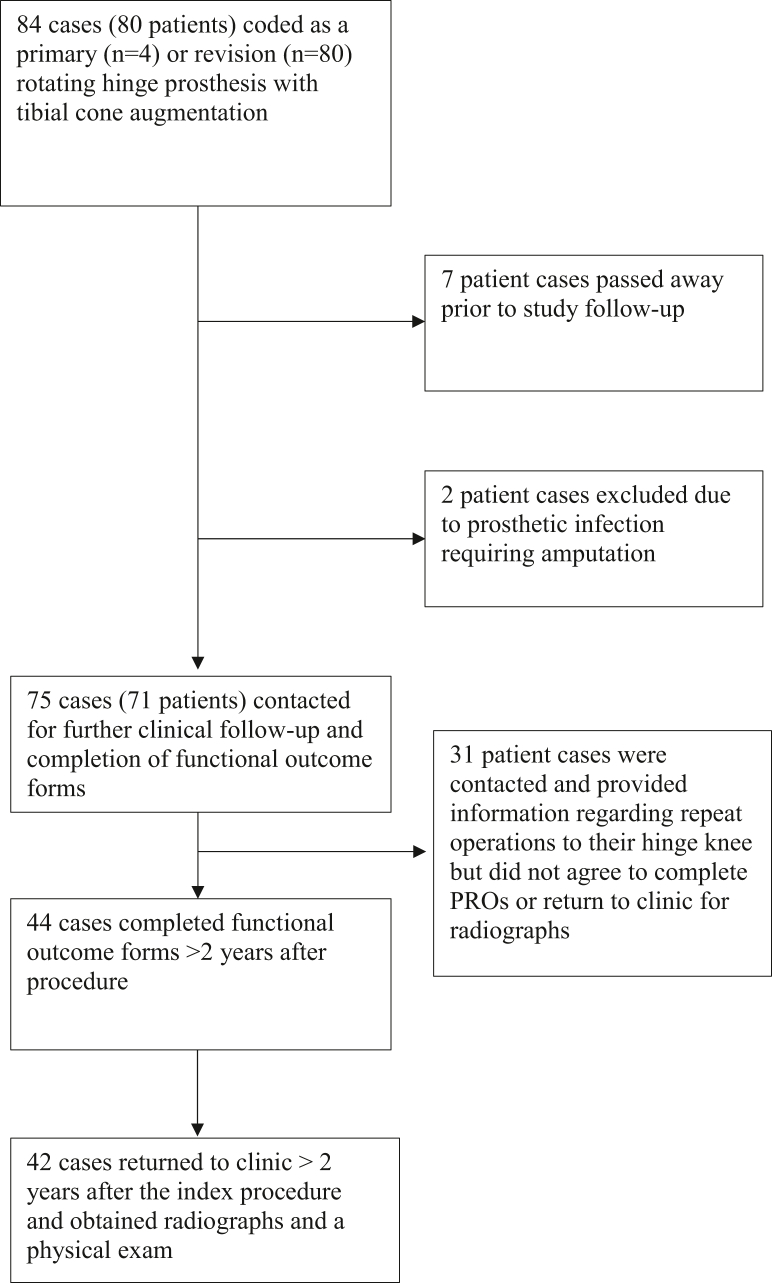
Flow chart demonstrating the distribution of cases as they were retrospectively reviewed and prospectively contacted for clinical follow-up.

Patient participation, coordination for clinic visits, and completion of PROs were followed via phone call. A total of 84 RHK with MTC augmentation cases met inclusion criteria. Four surgeons contributed to this case total. Bilateral cases were noted in 4 patients. Of the 84 cases included in the study, 4 were primary RHK and 80 were revision RHK. In cases where the use of a hinge prosthesis was performed as a revision (n = 80), the demographic of removed implant was primary hinge (8%), primary TKA (35%), and revision components or spacers (57%). Death records reported 7 deceased patients since their last follow-up. Further review demonstrated 2 patients who experienced persistent prosthetic joint infection requiring amputation, leaving a total of 71 patients (75 cases) to be contacted for study participation. If patients were unable to participate in clinical follow-up or complete PROs, they were asked over the phone if they had a subsequent surgery on their knee. Prospectively contacting patients along with retrospective chart review allowed for the construction of a cumulative incidence function (CIF) curve, with failure defined as a subsequent revision. Clinical radiographs and physical exams were obtained from 42 cases which was 56% of the potential cohort (Fig. 1). Functional outcome forms were collected from 44 cases, totaling 59% of the potential cohort (Fig. 1). Of the 44 cases with surgical and PRO data recorded, 2 were primary RHKs. One primary hinge knee was indicated for extreme stiffness status post multiligamentous reconstruction with a total range of motion of 30° before surgery. The second primary RHK was indicated for multidirectional native knee instability with 20° of recurvatum in a nonpolio case.

The full cohort (n = 84) ranged in age from 37-89 years (mean ± standard deviation(SD): 68.3 ± 10.6 years) and had an average body mass index of 34.4 ± 9.6 kg/m^2^. A total of 52 (61%) participants were women, 25 (30%) had diabetes, and 4 (5%) were current tobacco users. Patient demographics of those who completed PROs >2 years out from their index RHK were substratified and included in Table 1.

**Table 1:** Demographics of RHK arthroplasty cases with MTC augmentation who completed PROs >2 years after their procedure.

Measure	Patients completing PRO (n = 44)
Age (y)^a^	67.4 ± 9.0
Sex (n, % male)	17 (38.6)
BMI (kg/m^2^)^a^	34.4 ± 8.4
Diabetes (n)	8
Follow-up (y)^b^	3.82 [2.79, 5.14]

BMI, body mass index.

a Mean ± SD.

b Median (interquartile range).

The indications for the RHK-MTC systems are listed in Table 2. The most common indication was aseptic loosening with severe bone loss, followed by global instability. RHK system was collected and reported in Table 3. A combined 4 cases had a hinged implant with varying brand from the tibial cone (2 Stryker cones (Stryker, Kalamazoo, MI) paired with Zimmer hinged TKAs (Zimmer Biomet, Warsaw, IN) and 2 Stryker cones (Stryker) paired with DePuy hinge TKAs (DePuy, Warsaw, IN). The remaining 80 cases had a hinge implant and tibial cone from the same brand. All 84 cases had a tibial cone only, while 19% had a tibial and femoral cone.

**Table 2:** Indication for the index RHK arthroplasty cases with MTC augmentation for the entire cohort and the subset of cases with outcome data at > 2 years follow-up.

Indication	Entire cohort (n = 84)	Cohort with Outcome data >2 Y (n = 44)
Primary hinge knee	9	3
Periprosthetic joint infection	16	10
Instability	29	15
Aseptic loosening	25	15
Periprosthetic fracture	5	1

**Table 3:** System breakdown of RHK arthroplasty cases with MTC augmentation for the entire cohort and the subset of cases with outcome data at > 2 years follow-up.

Hinge system	Entire cohort (n = 84)	Cohort with Outcome data >2 Years (n = 44)
Smith and Nephew-Legion^a^	59	27
Stryker- Modular Rotating Hinge^b^	23	16
DePuy–S-ROM NOILES^c^	2	1

a Smith and Nephew, Memphis, Tennessee, USA.

b Stryker, Kalamazoo, MI, USA.

c DePuy, Warsaw, IN, USA.

Four different cementing techniques were used for the stems and condylar aspect of the hinge TKAs. In all 4 techniques the cones were press fit. Construct A was used in 75 cases. In construct A, the femoral stem and tibial stem were fully cemented with the tibial stem being cemented through the cone, back filling cement into the tibia after the cement restrictor and cone were placed. Construct B was used in 5 cases and utilized a hybrid technique for both the tibia and femoral components where the stems and cones were press fit and cement was packed only around the metaphyseal aspect of the implants. Construct C was utilized in 2 cases with fully cemented stems for the tibial implant and a hybrid technique for the femoral implant. Construct D was performed in the remaining 2 cases where a hybrid technique was used for the tibial implant and the femoral implant was fully cemented. Stryker Simplex Cement with Tobramycin (Antibiotic Simplex with Tobramycin, Stryker Howmedica Osteonics Corp., Mahwah, NJ) was utilized in all cases.

### Data analyses

Descriptive statistics were performed separately for the survival analysis cohort (n = 84) and the 2-year postoperative outcome cohort (n = 44). Participant characteristics, as well as PROs scores, were described using mean ± SD for continuous variables and frequency (percentage) for categorical variables. The CIF was used to estimate the probability of reoperation over time while accounting for the competing risk of death. Unlike Kaplan–Meier survival analysis, which treats censored individuals as if they remain at risk indefinitely, CIF correctly adjusts for the fact that death prevents the occurrence of reoperation. This approach provides a more accurate representation of the cumulative risk of reoperation in the presence of competing events. Analyses were completed using SAS statistical software version 9.4 (SAS Institute, Inc., Cary, NC).

## Results

### Survivorship and MTC structural stability

**Figure 2:**
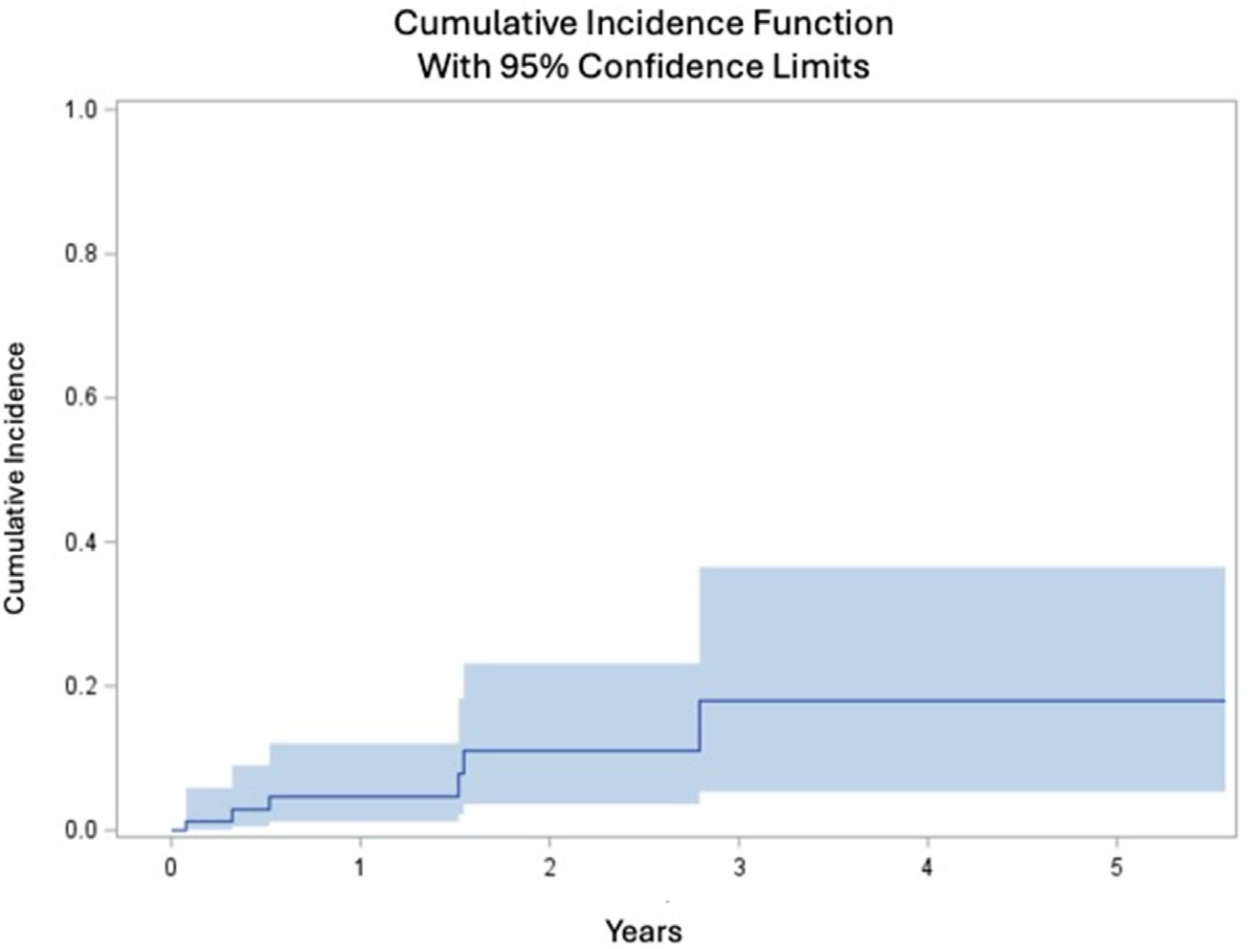
CIF curve for all-cause failure of the rotating hinge knee prostheses paired with MTCs.

The maximum time to failure was 2.8 years. Maximum follow-up for participants who did not experience failure was 5.48 years post-RHK among observations included in survival analyses (n = 84). The CIF analysis estimated risk of all-cause failure at 2.8 years to be 18 ± 8% (Fig. 2). The diagnoses for failed cases included infection (6 cases), mechanical failure (1 case), and persistent pain (1 case). Of the 6 infection failures, 5 of the RHKs were indicated in the setting of revision for previous periprosthetic joint infection (PJI). Infection cases were managed with debridement, antibiotics, and implant retention (4 cases) or above-knee amputation (2 cases). Patellar resurfacing was performed for the single case of persistent pain.

**Figure 3:**
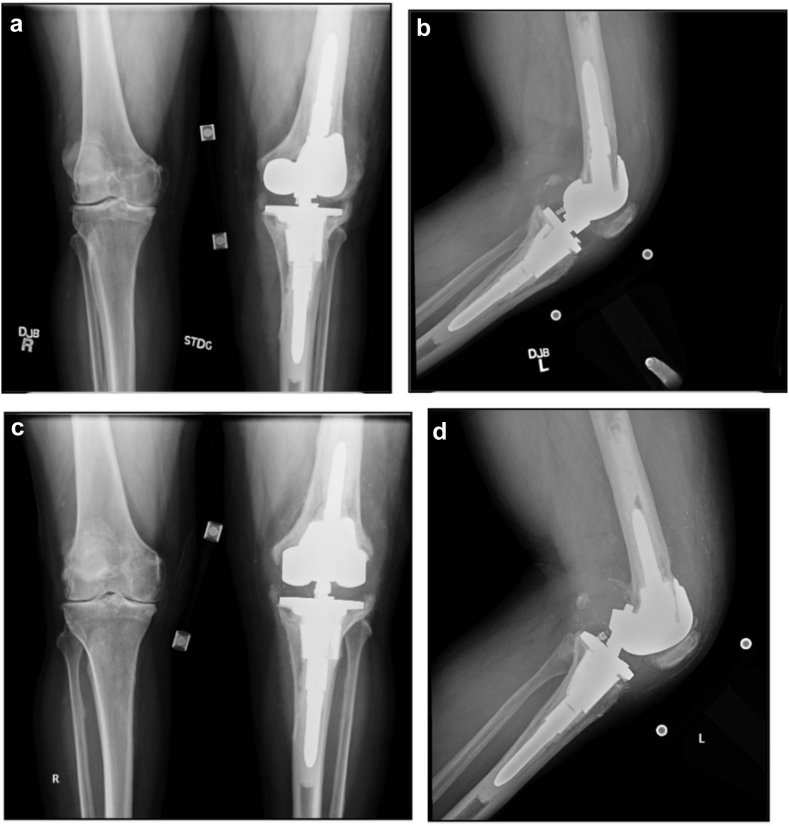
(a) Anteroposterior and (b) lateral radiographs of the single mechanical failure case secondary to aseptic loosening of the femoral component before the first revision. (c) Anteroposterior and (d) lateral radiographs of the previously revised RHK for mechanical failure before the second revision surgery which was indicated for a bent hinge mechanism.

When assessing survivorship of the subset of cases with clinical follow-up > 2 years (n = 42), no cases demonstrated radiographic or clinical evidence of loosening at the MTC-RHK interface with a median follow-up time of 3.82 years. The only case in this cohort requiring a subsequent operation was for mechanical failure. This case entailed 2 subsequent revisions (Fig. 3). The first was indicated for loosening of the femoral component requiring femoral only revision and the second revision being done for a broken hinge component for which the hinge and liner were replaced. The original indication for the RHK was revision of a prior PJI.

### Clinical outcomes

Clinical data were collected from 44 of the 84 (52%) cases beyond 2 years with a median follow-up of 3.82 years. The median knee active range of motion was 0°-120°. A total of 8 patients had an extensor lag of 10°-20°. No patients had an extensor lag greater than 20°. There were no cases with a known mechanism rupture at the time of the recorded physical exam. Mean ± SD PRO scores were 61.4 ± 18.7 for KOOS-JR, 47.9 ± 8.9 for PROMIS Mental Health, and 41.3 ± 8.7 for PROMIS Physical Health at 3.82-year follow-up. PASS was attained by 68% of follow-up cases and 75% reported moderate or greater improvement in quality of life since their RHK.

## Discussion

There is limited investigation regarding construct failure and PROs of a rotating hinge prosthesis when combined with a tibial cone. In the present study no cases demonstrated radiographic or clinical signs of loosening at the RHK–MTC interface, indicating a robust construct. We found an overall failure estimate of 18% after 2.8 years. The leading cause of failure in our study was attributed to PJI, 71% of which were originally indicated for revision of previously infected knees. Previous studies investigating RHK outcomes have reported failure rates of 10%-46% [6,12,17].

Cottino et al found an all-cause reoperation rate for their cohort of RHK to be roughly 10% at 2 years with low rates of aseptic loosening (4.5%) [17]. Aseptic loosening of both the femoral and tibial implants were noted in 1.7% (n = 6) of cases, isolated femoral component loosening was noted in 1% (n = 4) of their cohort while 0.8% (n = 3) of their cohort had only tibial component loosening. Like the present study, infection was the leading cause for further revision, with a prevalence of 35%. A subset of the cohort utilized tibial and femoral cones (28%) which was suspected to have a protective effect against aseptic loosening, although this was a statistically insignificant finding [17]. Smith et al noted an overall survivorship of a studied RHK cohort to be 54% at 4 years with infection being their highest mode of failure at a rate of 24% and aseptic loosening occurring at 8% [12]. Pour et al reported aseptic loosening as a mode of failure in roughly 9 % (n = 4) and infection in 7% (n = 3) of cases at 1.7 years follow-up [6]. Three of the 4 aseptic loosening failures were due to femoral implant loosening and 1 was due to tibial implant loosening [6]. In this study, the single case of persistent knee pain requiring patellar resurfacing aligns with a commonly recognized cause for reoperation with RHK. Bruce et al investigated long term RHK outcomes and recommended patellar resurfacing at the time of the index rotating hinge arthroplasty due to 8% of unresurfaced patellae requiring eventual revision [18].

Functional outcome measures are increasingly being used to help guide clinical management [19]. Patients in this study reported improved function after their hinge knee procedure with 75% of the respondents endorsing moderate improvement or better. When comparing our results to studies analyzing RHK outcomes, improvement in PROs is consistently noted [13,14,17]. Our study utilized the KOOS-JR outcome form to assess knee function in contrast to these other studies which utilized the Knee Society Score. KOOS-JR is a validated outcome form for primary and revision arthroplasty with high internal consistency and external validity [20,21]. The present study’s mean score of 61.4 represents modest knee function as a score of 100 is ‘perfect knee health’ and 0 is ‘complete disability’. In conjunction with this moderate KOOS-JR score, 68% of patients felt their knee was in an acceptable state after their RHK with MTC augmentation. Kearns et al found similar patient satisfaction with 72% of patients finding satisfaction with their RHK. These findings are not surprising given the complexity of the cases in this patient cohort and alternative options to a RHK often being knee arthrodesis or amputation.

The present study had several limitations. Retrospective analysis of medical records at a single institution was utilized to develop an all-cause failure analysis. Our tertiary facility receives complex referrals from across the region with patients often living hours away, making it difficult for all the patients to maintain consistent follow-up. Additionally, patients may have moved or were incapable of taking time off work to come back for radiographs and clinical evaluation for the study, limiting our cohort size. While this is the largest cohort of RHK with MTC that we know of, it is still a small number of cases. A comparison group was unable to be constructed in this study due to a lack of case numbers for RHK implants without MTC augmentation at our institution. Existing literature was relied upon and used for comparison as noted in the discussion. Additional studies with larger sample sizes and comparative groups are warranted to delineate the success of RHK when paired with MTC. Finally, our median follow-up time was just under 4 years. Four years is in the short term, and longer follow-up is needed to detect modes of structural failure.

## Conclusions

Combining RHK implants with a MTC is a durable construct with lower rates of aseptic loosening in the short term when compared to RHK implants without tibial cone augmentation previously reported in the literature. The present study’s results provide a preliminary report suggesting RHK when paired to a MTC may provide a protective effect against aseptic loosening of the tibial component while providing mostly satisfactory knee function and consistent self-reported improvement.

## Conflicts of interest

Nicolas Noiseuxis a paid consultant for Zimmer Biomet and receives research support from Smith & Nephew and DePuy as a principal investigator. The other authors declare there are no conflicts of interest.

For full disclosure statements refer to https://doi.org/10.1016/j.artd.2025.101724.

## CRediT authorship contribution statement

**Matthew Van Engen:** Writing – review & editing, Writing – original draft, Project administration, Methodology, Formal analysis, Data curation. **Taylor Den Hartog:** Writing – review & editing, Methodology, Formal analysis, Data curation. **Natalie Glass:** Writing – review & editing, Project administration, Methodology, Formal analysis, Data curation. **Nicolas Noiseux:** Writing – review & editing, Writing – original draft, Supervision, Project administration, Methodology, Investigation, Data curation, Conceptualization.

## Appendix A Supplementary data

Conflict of Interest Statement for Glass

Conflict of Interest Statement for Van Engen

Conflict of Interest Statement for Den Hartog

Conflict of Interest Statement for Noiseux
