# Supplementary material for: Two- to Six-year Assessment of Survivorship and Patient-Reported Outcomes in Rotating Hinge Knee Prostheses With Porous Tibial Cone Augmentation
Source: Arthroplast Today. 2025 Jun 2;33:101724. doi: 10.1016/j.artd.2025.101724 (PMC12167116; doi:10.1016/j.artd.2025.101724)
Supplement: Conflict of Interest Statement for Glass [file mmc1.pdf]

INDIVIDUAL CONFLICT OF INTEREST STATEMENT

American Association ofHip and Knee Surgeons

(Adopted from the American Academy of Orthopaedic Surgeons disclosure statement)

The following form must be filled out completelv and submitted bv each author (example. 6 authors. 6 forms). All items require a response. If there is no relevant disclosure for a qiven item, enter "None."

Manuscript Title 2-6 year Assessment of Survivorship and Patient Reported Outcomes in Rotating Hinge Knee Prosthesis with Porous Tibial Cone Augmentation

1.Royalties from a company or supplier (The following conflicts were disclosed)

None

2. Speakers bureau/paid presentations for a company or supplier (The following conflicts were disclosed)

None

3A. Paid employee for a company or supplier (The following conflicts were disclosed)

None

3B. Paid consultant for a company or supplier (The following conflicts were disclosed)

None

3C.Unpaid consultants for a company or supplier (The following conflicts were disclosed)

None

4. Stock or stock options in a company or supplier (The following conflicts were disclosed)

None

5. Research support from a company or supplier as a Principal Investigator (The following conflicts were disclosed)

None

6. Other financial or material support from a company or supplier (The following conflicts were disclosed)

None

7. Royalties, financial or material support from publishers (The following conflicts were disclosed)

None

8. Medical/Orthopaedic publications editorial/governing board (The following conflicts were disclosed)

None

9. Board member/committee appointments for a society (The following conflicts were disclosed)

None

Each author must siqn AND print or tvpe his/her name, date and submit a separate form

In addition, one BLINDED Conflict of Interest form (no author names used) should be submitted per manuscript with all author disclosures.

Natalie A. Glass 12/29/24

author disclosures.

Author Name (Print or Type) Author Signature Date
