# Supplementary material for: Two- to Six-year Assessment of Survivorship and Patient-Reported Outcomes in Rotating Hinge Knee Prostheses With Porous Tibial Cone Augmentation
Source: Arthroplast Today. 2025 Jun 2;33:101724. doi: 10.1016/j.artd.2025.101724 (PMC12167116; doi:10.1016/j.artd.2025.101724)
Supplement: Conflict of Interest Statement for Noiseux [file mmc4.pdf]

INDIVIDUAL CONFLICT OF INTEREST STATEMENT

American Association of Hip and Knee Surgeons

(Adopted from the American Academy of Orthopaedic Surgeons disclosure statement)

The following form **must be filled out completely and submitted by each author (example, 6 authors, 6 forms). All items require a response. If there is no relevant disclosure for a given item, enter "*None.*"**

Manuscript Title: 2-6 year Assessment of Survivorship and Patient Reported Outcomes in a Hinge Knee Prosthesis with Porous Tibial Cone Augmentation

1. Royalties from a company or supplier (The following conflicts were disclosed)
None

2. Speakers bureau/paid presentations for a company or supplier (The following conflicts were disclosed)
None

3A. Paid employee for a company or supplier (The following conflicts were disclosed)
None

3B. Paid consultant for a company or supplier (The following conflicts were disclosed)
Zimmer-Biomet

3C. Unpaid consultants for a company or supplier (The following conflicts were disclosed)
None

4. Stock or stock options in a company or supplier (The following conflicts were disclosed)
None

5. Research support from a company or supplier as a Principal Investigator (The following conflicts were disclosed)
Smith & Nephew
DePuy

6. Other financial or material support from a company or supplier (The following conflicts were disclosed)
None

7. Royalties, financial or material support from publishers (The following conflicts were disclosed)
None

8. Medical/Orthopaedic publications editorial/governing board (The following conflicts were disclosed)
None

9. Board member/committee appointments for a society (The following conflicts were disclosed)
None

Each author must sign AND print or type his/her name, date and submit a separate form

In addition, one BLINDED Conflict of Interest form (no author names used) should be submitted per manuscript with all author disclosures.

Author Name (Print or Type)	Author Signature	Date
**Nicolas Noiseux**	Nicolas O. Noiseux	12/29/24
